# Polyphenolic QTOF-ESI MS Characterization and the Antioxidant and Cytotoxic Activities of *Prunus domestica* Commercial Cultivars from Costa Rica

**DOI:** 10.3390/molecules26216493

**Published:** 2021-10-27

**Authors:** Mirtha Navarro-Hoyos, Elizabeth Arnáez-Serrano, Silvia Quesada-Mora, Gabriela Azofeifa-Cordero, Krissia Wilhelm-Romero, María Isabel Quirós-Fallas, Diego Alvarado-Corella, Felipe Vargas-Huertas, Andrés Sánchez-Kopper

**Affiliations:** 1 Bioactivity & Sustainable Development (BIODESS) Group, Department of Chemistry, Rodrigo Facio Campus, University of Costa Rica (UCR), San Pedro Montes Oca, San Jose 2060, Costa Rica; krissia.wilhelm@ucr.ac.cr (K.W.-R.); maria.quirosfallas@ucr.ac.cr (M.I.Q.-F.); luis.alvaradocorella@ucr.ac.cr (D.A.-C.); luis.vargashuertas@ucr.ac.cr (F.V.-H.); 2 Department of Biology, Costa Rica Institute of Technology (TEC), Cartago 7050, Costa Rica; earnaez@itcr.ac.cr; 3 Department of Biochemistry, School of Medicine, Rodrigo Facio Campus, University of Costa Rica (UCR), San Pedro Montes Oca, San Jose 2060, Costa Rica; silvia.quesada@ucr.ac.cr (S.Q.-M.); gabriela.azofeifacordero@ucr.ac.cr (G.A.-C.); 4 Centro de Investigación y de Servicios Químicos y Microbiológicos (CEQIATEC), Department of Chemistry, Costa Rica Institute of Technology (TEC), Cartago 7050, Costa Rica; ansanchez@itcr.ac.cr

**Keywords:** *Prunus domestica*, plum, UPLC, mass spectrometry, QTOF ESI-MS, polyphenols, procyanidins, flavonoids, antioxidant, antitumoral

## Abstract

There is an increased interest in plum research because of their metabolites’ potential bioactivities. In this study, the phenolic profiles of *Prunus domestica* commercial cultivars (Methley, Pisardii and Satsuma) in Costa Rica were determined by Ultra Performance Liquid Chromatography coupled with High Resolution Mass Spectrometry using a quadrupole-time-of-flight analyzer (UPLC-ESI-QTOF MS) on enriched phenolic extracts obtained through Pressurized Liquid Extraction (PLE) under acidic and neutral extraction conditions. In total, 41 different phenolic compounds were identified in the skin and flesh extracts, comprising 11 flavan-3-ols, 14 flavonoids and 16 hydroxycinnamic acids and derivatives. Neutral extractions for the skins and flesh from all of the cultivars yielded a larger number of compounds, and were particularly rich in the number of procyanidin trimers and tetramers when compared to the acid extractions. The total phenolic content (TPC) and antioxidant potential using the DPPH and ORAC methods exhibited better results for neutral extracts with Satsuma skins and Methley flesh, which showed the best values (685.0 and 801.6 mg GAE/g extract; IC_50_ = 4.85 and 4.39 µg/mL; and 12.55 and 12.22 mmol TE/g extract, respectively). A Two-Way ANOVA for cytotoxicity towards AGS gastric adenocarcinoma and SW620 colon adenocarcinoma indicated a significant difference (*p* < 0.05) for PLE conditions, with better results for neutral extractions, with Satsuma skin delivering the best results (IC_50_ = 60.7 and 46.7 µg/mL respectively) along with Methley flesh (IC_50_ = 76.3 and 60.9 µg/mL, respectively). In addition, a significant positive correlation was found between TPC and ORAC (r = 0.929, *p* < 0.05), as well as a significant negative correlation (*p* < 0.05) between TPC and cytotoxicity towards AGS and SW620 cell lines (r = −0.776, and −0.751, respectively). A particularly high, significant, negative correlation (*p* < 0.05) was found between the number of procyanidins and cytotoxicity against the AGS (r = −0.868) and SW620 (r = −0.855) cell lines. Finally, the PCA clearly corroborated that neutral extracts are a more homogenous group exhibiting higher antioxidant and cytotoxic results regardless of the part or cultivar; therefore, our findings suggest that PLE extracts under neutral conditions would be of interest for further studies on their potential health benefits.

## 1. Introduction

The bioactive properties of polyphenols have generated much interest in recent years, increasing the consumption of drinks and foods rich in these compounds, and generating greater attention for their research. One of the major interests of these compounds relates to their antioxidant activity, i.e., the prevention of oxidative stress measured by radical activity and its related health benefits [1].

There are reports on the beneficial effects of polyphenols in long-term health protection based on data collected in population studies and clinical trials [2]. For instance, polyphenols offer protection against reactive oxygen and nitrogen radical species, UV light, and pathogens, but also reduce the risk of chronic and degenerative diseases, cardiovascular problems, and cancer [3]. These outstanding properties are still in intense study due to new evidence suggesting that their radical scavenging activities are involved in their metabolic modulation properties in vivo [4].

Plums are fruits rich in polyphenols such as proanthocyanidins, flavonoids, hydroxycinnamic acids and coumarins, and in some countries these fruits—which exhibit a great diversity in shape, size, appearance and taste—are used as medicinal products [5,6]. The global annual production of plums is approximately 11,000,000 tons, and they are marketed worldwide, with the highest demand in Europe, North America and Japan [7].

Although attention has increased towards the ways in which polyphenolic extraction methods and conditions may influence the final composition of samples [8], the reports for plums mainly focus on one type of polyphenols, such as acids or flavonoids. In addition, the reports are often performed for the fruit as a whole, or in a selected fruit part, for instance the flesh or skins [5] [7]. Based on preliminary findings showing the potential of polyphenolic contents in the main Costa Rican commercial plum cultivar [9], we aimed to evaluate the skins and flesh of different plum commercial cultivars under different extraction conditions in order to study their polyphenols and their biological activities.

Therefore, the aim of the present work was to obtain enriched polyphenolic extracts of *P. domestica* commercial cultivars in Costa Rica, and to characterize them through Ultra Performance Liquid Chromatography coupled with High Resolution Mass Spectrometry (UPLC-ESI-MS). Furthermore, the evaluation of the total polyphenolic contents and antioxidant activity using 2,2-diphenyl-1-picrylhidrazyl (DPPH) and oxygen radical absorbance capacity (ORAC) methods, as well as the cytotoxic activity (MTT) on adenocarcinoma AGS gastric cell lines and adenocarcinoma SW620 colon cell lines, was also carried out with the different extracts.

## 2. The Results and Discussion

### 2.1. Phenolic Yield and Total Phenolic Contents

The total phenolic content (TPC) was evaluated separately for the skin and flesh from the three main Costa Rican plum cultivars, namely Methley, Satsuma and Pisardii, using Pressurized Liquid Extraction (PLE) under neutral and acidic aqueous methanolic conditions, as described in the Materials and Methods section. The results for the TPC are summarized in Table 1 for the twelve extracts. The results indicated that neutral PLE extractions showed higher TPC content than PLE acid extractions for all of the samples. When comparing among the cultivars, Methley flesh and Satsuma skins showed the highest TPC value (801.6 mg gallic acid equivalents (GAE)/g extract and 685 mg GAE/g extract respectively) using PLE under neutral conditions, while Methley cultivar skin and flesh extracts exhibited the highest values in acidic PLE conditions (539.3 and 567.7 mg GAE/g extract respectively). In turn, Pisardii flesh and skin extracts under acid conditions showed the lowest values (319 and 438.5 mg GAE/g extract, respectively) among all twelve extracts.

In order to assess the influence of the fruit part and type of extraction on the TPC, a two-way Analysis of Variance (ANOVA) was carried out. The results showed no significant differences for the fruit part; however, there is significant difference (*p* < 0.05) regarding the extraction method, with neutral PLE extraction yielding better TPC values for both fruit parts. Previous studies have indicated a dependence on the nature of the polyphenols present in the matrix, e.g., with neutral conditions being more favorable because of the structural stability of flavan-3-ols [1], while acidic conditions give better results for anthocyanin in acidic conditions [8]. On the other hand, previous literature reports on the total phenolic contents for plums indicated values for the whole fruits varying between 18.82 to 25.58 mg GAE/g DW for French cultivars [10], and from 1.38 to 7.61 mg GAE/g FW [11,12] for Japanese and European cultivars. Other studies showed TPC values ranging between 85.2 and 773 mg GAE/100 g FW [13,14,15] for the skins of Spanish and Romanian plum cultivars, while our results indicate values between 161 and 631 mg GAE/100 g FW, which are within the range when comparing them with these studies. In respect to the flesh, previous reports have shown TPC values varying from 36.4 to 180.8 mg GAE/100 g FW for European cultivars [13,14], while Costa Rican plum values are higher, ranging from 91.1 to 323 mg GAE/100 g FW. Finally, a report for plum leaf extracts from Romanian cultivars showed values from 82.84–139.67 mg GAE/g extract [16], which is lower than our results for the skin and flesh of Costa Rican plum fruits extracted under neutral PLE conditions ranging between 319.9 and 801.6 mg GAE/g extract.

### 2.2. Profile by UPLC-ESI-MS/MS Analysis

**Figure 1:**
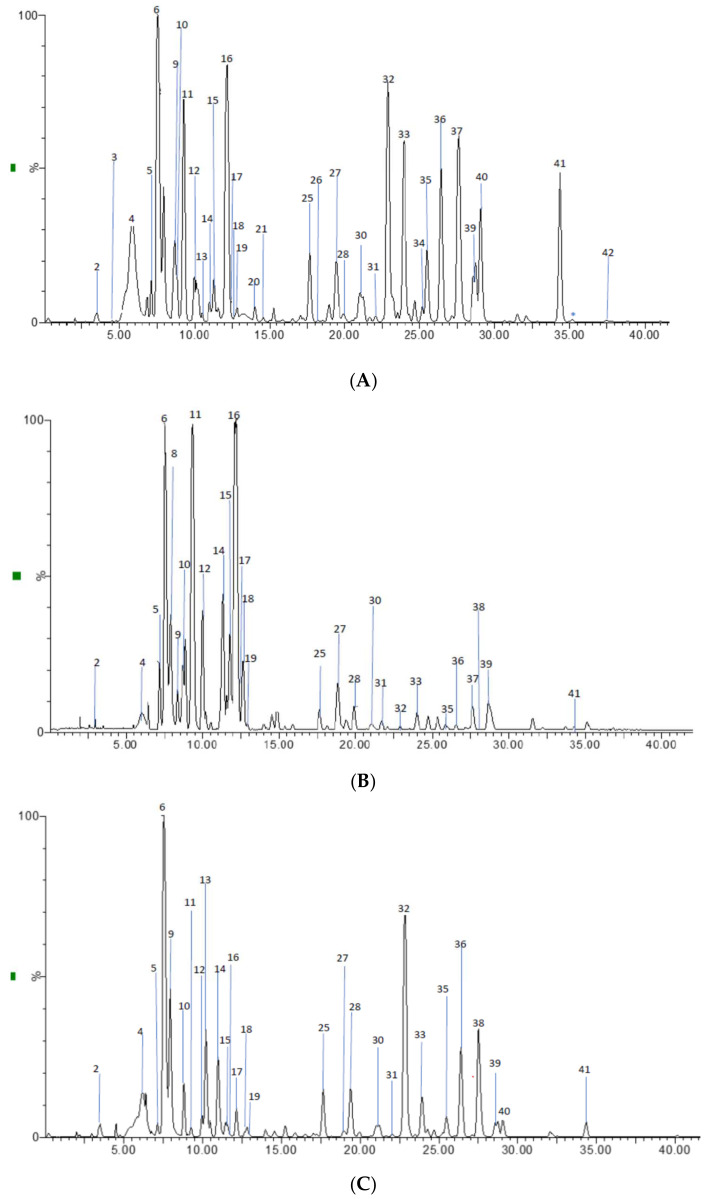

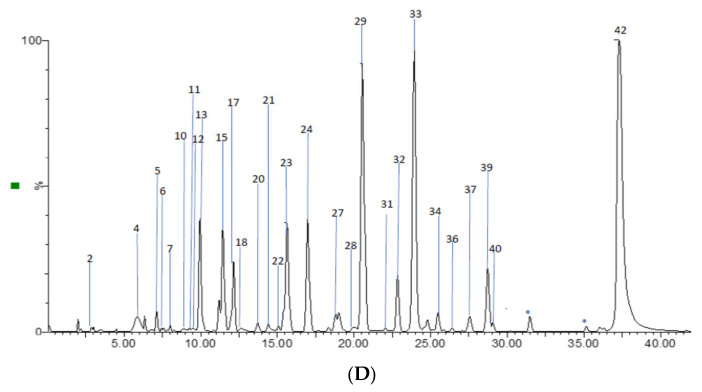
HPLC chromatograms of: (**A**) Methley skin, (**B**) Methley flesh, and (**C**) Pisardii flesh extracts (PLE, neutral conditions), and (**D**) Satsuma skin extract (PLE, acid conditions) in a Phenomenex Luna RP18 C-18 column (150 mm × 4.6 mm × 4 µm) using a Xevo G2-XS QTOF Mass spectrometer (Waters™, Manchester, UK) in a mass range from 100 to 1500 amu.

The UPLC-ESI-QTOF MS analysis described in the Materials and Methods section allowed us to identify 41 phenolic compounds in the skin and flesh extracts from the main Costa Rican commercial cultivars, including nine procyanidin oligomers (dimers, trimers and tetramers), the two flavan-3-ol monomers, 14 flavonoids (kaempferol and quercetin derivatives), and 16 hydroxycinnamic acids and related compounds (HCA). Figure 1 shows the chromatograms of the different compounds, and Table 2 summarizes the results of the identification analysis.

#### 2.2.1. Hydroxycinnamic Acids and Derivatives

**Figure 2:**
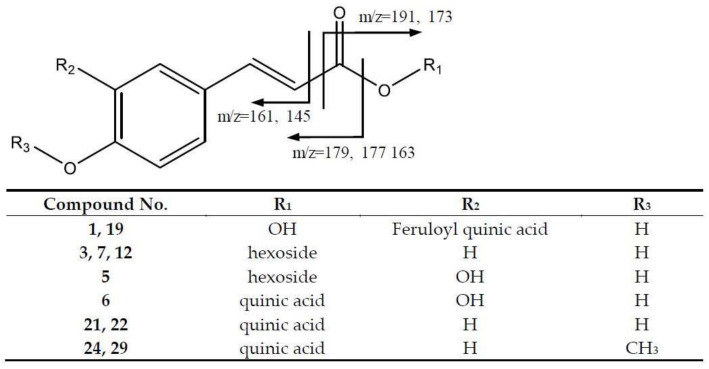
Hydroxycinnamic acid derivatives’ structures and main fragments.

In the first group of compounds, a series of 4-hydroxiccinamic acid derivatives were found (Figure 2). For instance, peaks 24 (Rt = 16.99 min) and 29 (Rt = 20.55 min), which correspond to methyl-coumaroylquinic acid at *m*/*z* 351.10.82 (C_17_H_19_O_8_) with a main fragment at *m*/*z* 177 due to the loss of quinic acid. Peaks 21 (Rt = 14.78 min) and 22 (Rt = 15.29 min) with [M-H]^−^ at *m*/*z* 337.0919 (C_16_H_17_O_8_) were assigned to p-coumaroylquinic acid [5], with a main fragment at *m*/*z* 173 that corresponds to the quinic acid fragment with the loss of water.

In addition, peaks 1 (Rt = 2.89 min) and 19 (Rt = 12.80 min), with [M-H]^−^ at *m*/*z* 529.1365 (C_26_H_25_O_12_), were identified as caffeoyl feruloylquinic acid isomers, following the fragmentation pattern [17] shown in Figure S1. The main fragments were found at *m*/*z* 353 [caffeoylquinic acid-H]^−^, 367 [feruloylquinic acid-H]^−^, 191 [quinic acid-H]^−^, and 179 [caffeoyl-H]^−^. In turn, peak 6 (Rt = 7.56 min) corresponds to caffeoylquinic acid with a [M-H]^−^ at *m*/*z* 353.0809 (C_16_H_17_O_9_), with MS2 fragments 191 [quinic acid-H]^−^ and 145 due to the loss of CO_2_ from the quinic acid ion. Peak 5 (Rt = 7.13 min) shows [M-H]^−^ at *m*/*z* 341.0872 (C_15_H_17_O_9_), corresponding to caffeoyl-hexoside, with the main fragments at *m*/*z* 161 [M-H-180]^−^ due to the loss of the hexose.

Peaks 13 (Rt = 10.23), 15 (Rt = 11.45) and 23 (RT = 15.65) corresponded to feruloylquinic acid isomers at *m*/*z* 367.1021 (C_9_H_5_O_3_); the peaks showed fragment ions at *m*/*z* 193 [ferulic acid-H]^−^, *m*/*z* 191 [quinic acid-H]^−^, and *m*/*z* 173 [quinic acid-H_2_O–H]^−^, which correspond to the fragmentation reported for the compound [18]. Additional hydroxycinnamic acid derivatives (Figure 2) were found in peaks 3 (Rt = 4.42 min), 7 (Rt = 7.96) and 12 (Rt = 9.98 min), with [M-H]^−^ at *m*/*z* 325.0917 (C_15_H_17_O_8_) being identified as coumaroyl-hexoside isomers, due to the fragments at *m*/*z* 163 [coumaric acid-H]^−^ and 145 [coumaric acid-H-H_2_O]^−^.

Peaks 25 (Rt = 17.44) and 31 (Rt = 21.03) (Figure S2), were identified as di-O-acetyl-O-p-coumaroylsucrose molecules with *m*/*z* at 571.1655, (C_25_H_31_O_15_) and main fragments at *m*/*z* 553 [M-18-H]^−^ due to the loss of water, 529 [M-42-H]^−^ because of acetyl loss, and 487 [M-84-H]^−^ due to the loss of both acetyl moieties [19].

Finally, it should be noted that peak 2 (Rt = 3.08) corresponds to quinic acid at *m*/*z* 191.0554 (C_7_H_11_O_6_), with a main fragment at *m*/*z* 173 [M-H-18]^−^ due to the loss of H_2_O.

#### 2.2.2. Flavonoids

Among the flavonoid derivatives, peak 42 (Rt = 37.25 min) corresponds to quercetin, showing a negative molecular ion peak [M-H]^−^ at *m*/*z* 301.0353 (C_15_H_9_O_7_), and the main fragment ions [20] are at *m*/*z* 179 and 151, which came from the retrocyclization pathway (Figure S3) [21]. Other fragments were found at *m*/*z* 257, representing the loss of CO_2_; *m*/*z* 283 [M-18-H]- due to loss of water; *m*/*z* 273 [M-28-H]-, owing to the loss of CO; and *m*/*z* 255 [M-18-28-H]- because the loss of water and CO were also observed.

Peak 4 (Rt = 5.89 min) with [M-H]^−^ at *m*/*z* 447.0913 (C_21_H_19_O_11_) was assigned as kaempferol hexoside, with a main fragment at *m*/*z* 285. Peaks 18 (Rt = 12.57 min) and 39 (Rt = 28.68 min) with [M-H]^−^ at *m*/*z* 447.0913 (C_21_H_19_O_11_) were designated as quercetin-deoxyhexoside isomers. Peaks 35 (Rt = 25.95 min) and 37 (Rt = 27.50 min) were elucidated based in the fragment of the aglycone due to the loss of glycosides, and had [M-H]^−^ at *m*/*z* 433.0769 (C_20_H_17_O_11_), corresponding with isomers of quercetin-pentoside [22].

**Figure 3:**
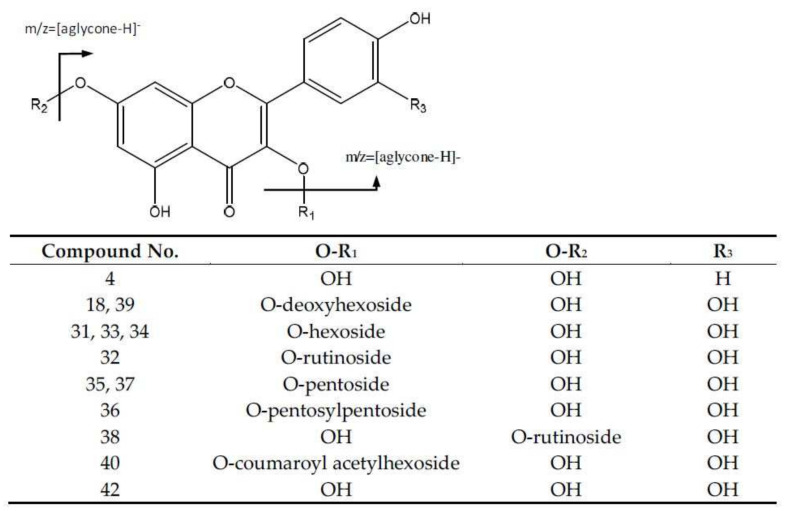
Flavonoids’ structure and main fragments.

Peaks 31 (Rt = 21.95 min), 33 (Rt = 23.89 min) and 34 (Rt = 25.46 min) with [M-H]^−^ at *m*/*z* 463.0875 (C_21_H_19_O_12_) correspond to quercetin-hexoside isomers, giving rise to the fragment at *m*/*z* 301 [M-162-H] after losing a hexose unit [23]. Peaks 32 (Rt = 22.86 min) and 38 (Rt = 27.86 min) with [M-H]^−^ at *m*/*z* 609.1488 (C_27_H_29_O_16_) were identified as quercetin-rutinoside. In turn, peak 36 (Rt = 26.24 min) had a [M-H]^−^ at *m*/*z* 565.1184 (C_25_H_25_O_15_), and was identified as quercetin-pentosyl-pentoside (Figure 3).

Peak 40 (Rt = 29.09 min) with [M-H]^−^ at *m*/*z* 505.0974 (C_23_H_21_O_13_) was identified as quercetin-acetylhexoside, with fragments at *m*/*z* 301 [M-162-H]^−^ and *m*/*z* 300 [M-163-H]^−^. Finally, peak 41 was tentatively identified as quercetin 3-O-p-coumaroylacetylhexoside, with a deprotonated molecular ion peak at *m/z* 651.1553 (C_32_H_27_O_15_), and fragment ions at *m/z* 505 [M-146-H]^−^ and 301 [M-146-204-H]^−^, corresponding to the losses of coumaroyl and acetylhexosyl [24] moieties.

#### 2.2.3. Flavan-3-ols Derivatives

**Figure 4:**
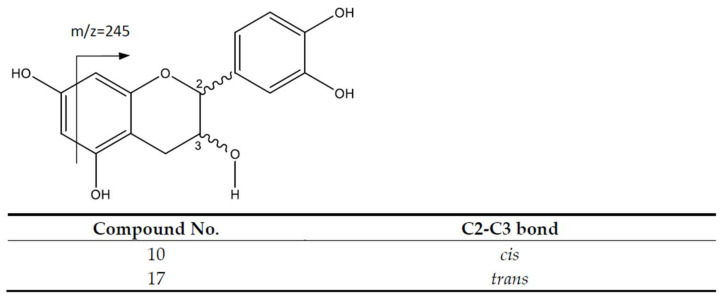
Flavan-3-ol monomer’s structure and main fragments.

The third group of compounds are flavan-3-ols, corresponding to monomers and procyanidin oligomers, including dimers, trimers and one tetramer. The flavan-3-ol monomers catechin and epicatechin (Figure 4) were assigned to peaks 10 (Rt = 8.81 min) and 17 (Rt = 12.16 min), showing [M-H]^−^ at *m*/*z* 289.0704, with main fragments at *m*/*z* 245 [M-42-H]^−^ because of the loss of C_2_H_4_O due to the retro-Diels-Alder fission (RDA) of ring A [25].

**Figure 5:**
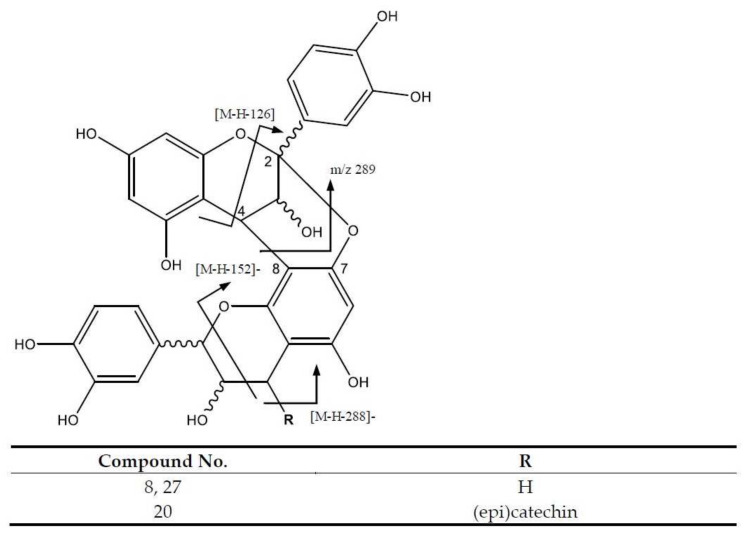
Procyanidin’s A-type structure and main fragments.

On the other hand, several procyanidin oligomers were identified, which can follow different fragmentation pathways—including Retro Diels-Alder (RDA), Heterocyclic Ring Fission (HRF) with the loss of phluoroglucinol (126 Da), and the Quinone–methide (QM) pathway with fragmentation between (epi)catechin units—to yield one of two different QM ions. Figure 5 illustrates this for procyanidin A-type dimers, of which the (epi)catechin units are linked through C2-O-C7 and C4-C8 bonds [26].

Peak 8 (Rt = 8.05 min) and peak 27 (Rt = 18.74 min) were tentatively identified as procyanidin A dimers showing [M-H]^−^ at *m*/*z* 575.1185 (C_30_H_23_O_12_), with a main fragment at *m*/*z* 449 [M-H-126]^−^ corresponding to the elimination of a phloroglucinol molecule along the HRF pathway. Furthermore, *m*/*z* 423 [M-H-152]^−^ is due to RDA cleavage, and *m*/*z* 289 and *m*/*z* 285 are from QM fission [27] (Figure S4). In turn, peak 20 (Rt = 13.71 min) was tentatively assigned to a procyanidin A trimer showing a [M-H]^−^ at *m*/*z* 863.1790 (C_45_H_35_O_18_), with fragment ions at *m*/*z* 575 and *m*/*z* 287 due to QM cleavage, and a fragment at *m*/*z* 711 [M-H-152]^−^ from the RDA fission [26].

**Figure 6:**
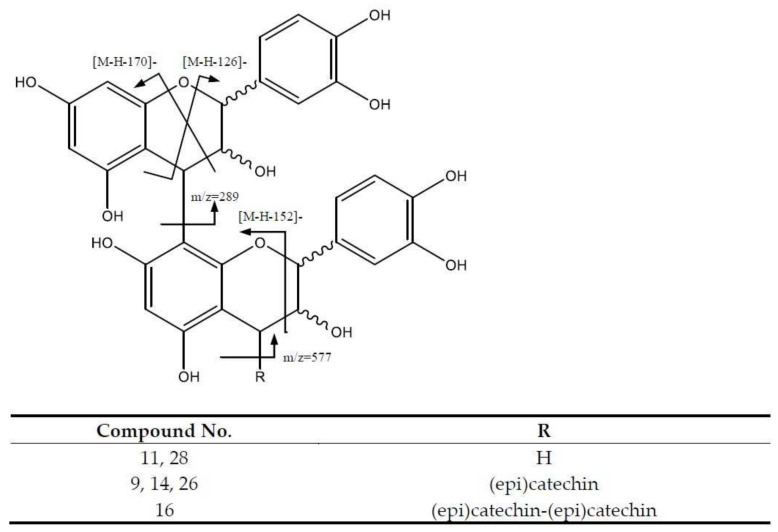
Procyanidin’s B-type structure and main fragments.

In addition, several B-type procyanidin dimers were identified (Figure 6). For instance, B-type dimers such as peaks 11 (Rt = 9.27 min) and 28 (Rt = 19.89 min) show [M-H]^−^ at *m*/*z* 577.1344 (C_30_H_25_O_12_); the major fragments at *m*/*z* 559 [M-H-18]^−^ originate from water loss. The fragment ions at *m*/*z* 425 [M-H-152]^−^ and 407 [M-H-170]^−^ come from RDA cleavage, while the ions at *m*/*z* 287 and *m*/*z* 289 originate from the QM pathway, and the fragment at m/z 451 corresponds to the loss of phloroglucinol (126 Da) via the HRF pathway (Figure S5) [26].

On the other hand, peaks 9 (Rt = 8.28 min), 14 (Rt = 11.25 min) and 26 (Rt = 18.28 min) with [M-H]^−^ at *m/z* 865.1993 (C_45_H_37_O_18_) were tentatively identified as procyanidin B-type trimers, with main fragments at m/z 695 [M-H-170]^−^, 713 [M-H-152]^−^ and 739 [M-H-126]^−^. In addition, the QM cleavage of the upper interflavanoid bond produced ions at *m*/*z* 287 and *m*/*z* 577, whereas the cleavage of the lower interflavanoid bond formed ions at *m*/*z* 289 and *m*/*z* 575 [27].

Finally, peak 16 (Rt = 11.86 min), with a [M-H]^−^ at *m*/*z* 1153.2579 (C_60_H_49_O_24_), was tentatively assigned to a procyanidin B-type tetramer with fragment ions for QM cleavage observed at *m*/*z* 287, 289, 575, 577 and 865. Furthermore, the fragment at *m*/*z* 1027 [M-H-126]^−^ corresponds to the neutral loss of phloroglucinol (Da 126) along the HRF fission pathway, and the fragments at *m*/*z* 1001 [M-H-152]^−^ and *m*/*z* 983 [M-H-170]^−^ are formed due to the RDA cleavage and a consecutive loss of water [26].

Regarding the total number of polyphenols in *P. domestica* commercial cultivars from Costa Rica, 306 compounds were found (Table S1), including 84 procyanidin oligomers, 102 flavonoids, and 120 hydroxycinnamic acids and related derivatives (HCA). The Methley and Satsuma cultivars showed the greatest numbers for both parts and extraction methods, with 110 and 102 compounds, respectively, while the Pisardii cultivar exhibited the lowest number, with 94 compounds.

Concerning the skin extracts, the Methley and Satsuma cultivars presented a similar number of compounds, which were higher than the Pisardii cultivar in both acid and neutral extractions, with flavonoids and HCA as the more abundant groups. In sum, the acidic and neutral skin extractions yielded 175 compounds, comprising 67 HCA, 65 flavonoids, and 43 flavan-3-ol monomers and procyanidin oligomers. For the flesh samples, the results showed a greater number of compounds in the neutral PLE conditions with respect to acidic PLE conditions for all of the cultivars. The neutral extraction of Satsuma showed the greatest number of procyanidin oligomers, while Pisardii flesh showed a greater number of HCA derivatives, especially in the acid extraction. In sum, the acid and neutral flesh extractions yielded 131 compounds, including 53 HCA, 37 flavonoids, and 41 flavan-3-ol monomers and procyanidin oligomers.

Finally, comparing the extractions, neutral PLE conditions showed more compounds than acidic PLE ones, with 172 compounds under neutral conditions versus a total of 134 compounds in acidic extractions. Neutral extractions for both skin and flesh are particularly abundant in procyanidins when compared to acid extractions, and are further particularly rich in the number of procyanidin trimer and tetramer oligomers (Table S1).

When comparing with literature reports, our results show a larger number of compounds than those reported for whole plum fruits from Egypt, USA, Serbia and Romania [16,28,29,30] in flavan-3-ols and HCA, as well as similar results in flavonoids to those found for Egyptian cultivars [29]. Within studies focused on plum skins, our results show a greater number of compounds than cultivars from the USA and Germany [7,31]. In the case of plum flesh, our findings indicate a similar number of compounds to those reported in German cultivars [5], especially for HCA, and higher than those reported for US cultivars [31].

### 2.3. Antioxidant Activity

The DPPH radical scavenging activity and Oxygen Radical Absorbance Capacity (ORAC) results for the twelve extracts from Costa Rican plum commercial cultivars are summarized in Table 3. Among the neutral PLE extractions for the DPPH assay, Methley flesh (IC50 = 4.39 μg/mL) and Satsuma cultivar skins (IC50 = 4.85 μg/mL respectively) showed the highest antioxidant capacity. In PLE acid extractions, Satsuma skin yielded the highest value (IC_50_ = 6.73 μg/mL), followed by Methley flesh (IC50 = 6.81 μg/mL). In turn, the lowest value of the three cultivars was found for Pisardii skin and flesh (IC_50_ = 12.72 μg/mL and IC_50_ 12.44 μg/mL) for acid extractions.

In order to evaluate the antioxidant activity in a comparative way, we used Trolox as a reference antioxidant agent. Our DPPH IC_50_ values for the twelve plum extracts were calculated as described in the Material and Methods section, using the DPPH IC_50_ obtained for Trolox (5.62 μg TE/mL). For instance, reports from the literature indicate values ranging from 1.83–3.5 mmol TE/g extract [16] and 23.09–27.72 mg TE/g dry material [10] for plum leaves and whole fruits from Romanian and French cultivars, respectively. Our results indicate higher DPPH values for plum fruit skin and flesh, ranging between 3.7 and 4.2 mmol TE/g extract, and 32.2 and 36.5 mg TE/g dry material, respectively.

In respect to the ORAC evaluation, neutral PLE extractions showed, once more, the best results when compared to acidic PLE conditions. Similarly to the DPPH results, ORAC showed that Satsuma skin extract under neutral PLE conditions holds the highest antioxidant values (12.55 mmol TE/g extract), along with Methley flesh (12.22 mmol TE/g extract). In addition, in an analogous way to DPPH, Pisardii skin and flesh under acidic PLE extraction showed the lowest values (7.45 and 7.04 mmol TE/g extract) among all twelve results. Compared with the reports from the literature, studies indicate values varying from 5.05 to 6.0 mmol TE/g extract, and 16.77 to 33.90 μmol TE/g fresh weight for Australian and Romanian cultivars, respectively [32,33], while our results show higher values ranging from 7.04–12.55 mmol TE/g extract and 36.17–47.78 μmol TE/g fresh weight.

**Figure 7:**
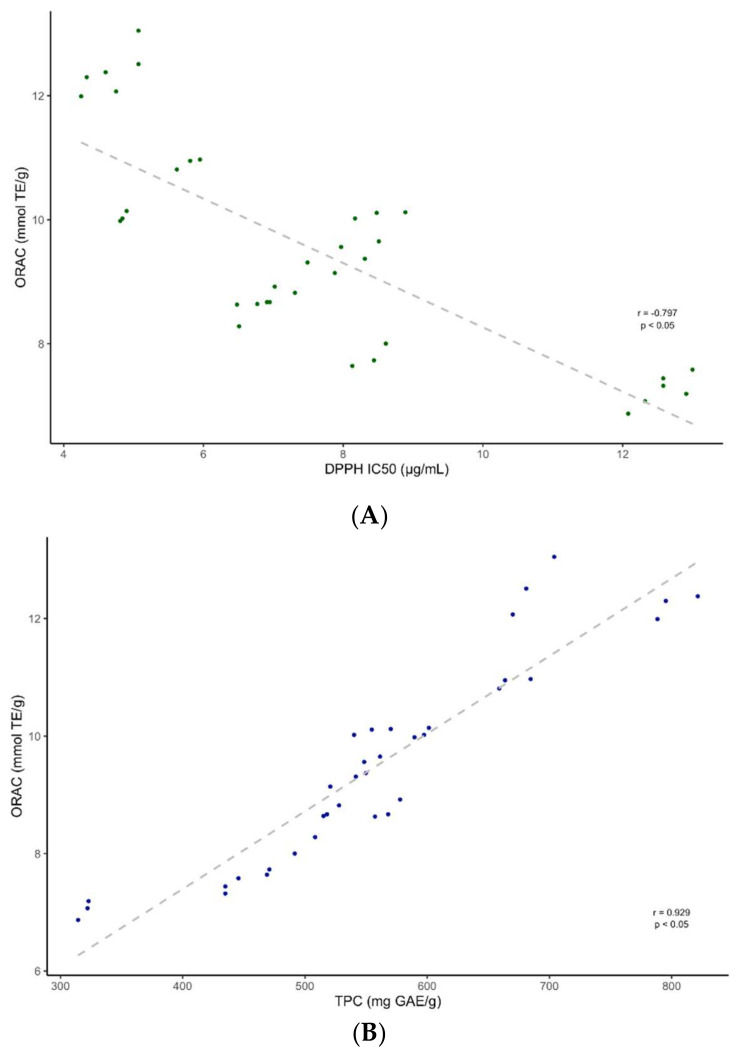

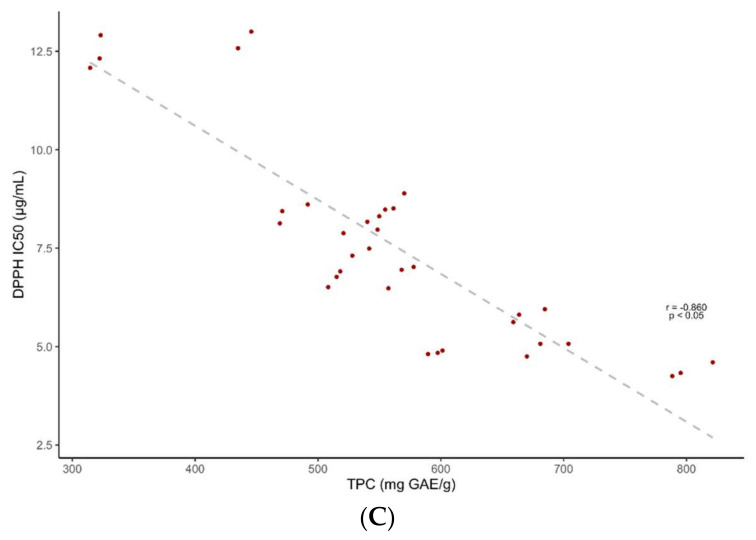
Correlation between (**A**) Total Phenolic Contents (TPC) and DPPH antioxidant scavenging values, (**B**) TPC and ORAC antioxidant capacity values, and (**C**) DPPH and ORAC values.

A two-way ANOVA analysis was performed to evaluate the influence of the two factors, i.e., the fruit part and PLE extraction conditions. In respect to DPPH, the results showed no significant difference for the fruit part or for the extraction conditions. In turn, the ORAC results showed a significant difference (*p* < 0.05) for the extraction method, with neutral PLE conditions rendering better results when compared to the acidic PLE extraction. Finally, the ORAC values indicated no significant difference for the fruit part. As shown in Figure 7, a correlation analysis was carried out between the TPC (Table 1), DPPH and ORAC values.

The findings indicated a significant positive correlation (*p* < 0.05) between the TPC and ORAC antioxidant results (*r* = 0.929), as well as a significant negative correlation (*p* < 0.05) between the TPC and DPPH values (*r* = −0.860). Therefore, our results align with previous studies reporting a correlation between the total polyphenolic contents and antioxidant results [34,35]. Finally, our findings indicate a negative correlation (*p* < 0.05) between the DPPH and ORAC values (R = −0.797), which were in agreement with previous studies [36].

A correlation analysis was also performed between the antioxidant values (Table 3) and the presence of polyphenolic groups of the compounds in the twelve extracts. The results indicated no significant correlation (*p* < 0.05) for flavonoids or HCA, which would explain the fact that although HCA constituted the largest number of skin acidic extracts, it seemed to have a low influence in the antioxidant activity.

In turn, a significant negative correlation (*p* < 0.05) was found between procyanidins and DPPH radical scavenging (*r* = −0.790), as well as a significant positive correlation between procyanidins and ORAC antioxidant capacity (*r* = 0.828), indicating that samples with a predominance of procyanidins, such as skin and flesh extracts under neutral conditions, would yield a higher antioxidant capacity (Table 4). This is in agreement with previous reports on procyanidins [37]; furthermore, the influence of a higher number of oligomers such as procyanidin trimers and tetramers would account for greater antioxidant values, as published earlier on procyanidin oligomers’ influence in antioxidant activities [38].

### 2.4. Cytotoxicity

**Figure 8:**
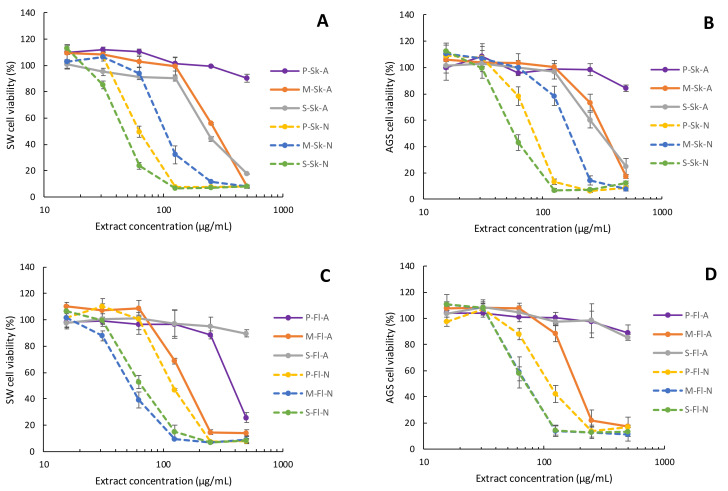
Cytotoxicity dose-response curves of the plum extracts on AGS and SW620 tumor cell lines. The results are presented as the mean ± SE of three independent experiments. (**A**) Skin samples in SW620 cells. (**B**) Skin samples in AGS cells. (**C**) Flesh samples in SW620 cells. (**D**) Flesh samples in AGS cells. Sample names: C-F-E: Cultivar-Fruit Part-Extraction. Cultivars: M (Methley), P (Pisardii), S (Satsuma). Fruit part: Sk (skin), Fl (flesh). Extraction: A (acidic PLE acid conditions), N (neutral PLE conditions).

Table 4 summarizes the IC50 values for the cytotoxic effect of *P. domestica* extracts on three different cell lines, namely AGS gastric adenocarcinoma cell lines, SW620 colon adenocarcinoma cells and Vero normal epithelial kidney cells. In addition, Figure 8 shows the cell viability dose–response curves for each extract.

A two-way ANOVA analysis of the IC50 values presented in Table 4 was performed to evaluate the influence of two factors: the fruit part and the extraction method. The findings indicate there is not a specific part of the fruit with a statistically improved cytotoxic activity; in some cultivars, the flesh showed better cytotoxicity, and in others it was the skin. On the other hand, the two-way ANOVA of the cytotoxic effect also reaffirmed a significant difference (*p* < 0.05) for extraction conditions, as was previously discussed in this paper for other parameters, such as the total phenolic content and antioxidant activities. The samples extracted in neutral PLE conditions yielded the best cytotoxicity, with values between 46.7 and 186 μg/mL, while the samples extracted in acidic PLE conditions displayed a less cytotoxic effect, with a range from 161 μg/mL to more than 500 μg/mL. As stated earlier, neutral extractions being more favorable could be related to the higher stability of the characterized polyphenols in these conditions [1].

The dose–response curves for each extract displayed in Figure 8 confirm the neutral extraction methodology as the best approach to obtain bioactive compounds. In fact, the four plots demonstrate a marked slope in the dose–response curves for the neutral extractions compared to the acid extractions. This pattern is independent of the cell line evaluated and the fruit part used for the polyphenol extracts. The comparison between the cytotoxic curves demonstrates that the Satsuma cultivar, for both the skin and flesh neutral extracts, as well as Methley flesh and Pisardii skin, present some of the best slopes in the dose–response curves in both tumoral cell lines tested.

Few previous publications reported the cytotoxic effect of *P. domestica* extracts in tumoral cell lines. A study that compared *P. domestica* cultivars displayed IC_50_ values ranging from 71.1 μg/mL to 144.5 μg/mL for a colorectal carcinoma cell line (HCT116); among them was a Methley cultivar grown in India, for which an IC50 of 85.9 μg/mL was reported [39]. In our assessment, Satsuma skins and Methley flesh extracts display IC_50_ values of 46.7 and 60.9 μg/mL, respectively, incubated with a colon adenocarcinoma cell line (SW-620), indicating a higher cytotoxic activity.

A significant negative correlation (*p* < 0.05) was observed between the TPC and IC50 cytotoxic values on AGS adenocarcinoma (*r* = −0.776), as well as TPC and IC50 cytotoxic values on SW620 adenocarcinoma (*r* = −0.751). These correlations suggest a role of the total content of polyphenolic compounds in the cytotoxic effect in tumoral cells. In addition, a specific correlation analysis was performed between the IC50 cytotoxic activity (Table 4) and the number of compounds of each type of polyphenol group identified (Table 2 and Table 3). The results showed no significant correlation with HCA or flavonoids for either the AGS or SW620 cell lines. However, the cytotoxic activity exhibited an important, significant negative correlation (*p* < 0.05) with the number of procyanidins for both cell lines, AGS (*r* = −0.868) and SW620 (*r* = −0.855). These specific r coefficient values are higher than the TPC r coefficients, suggesting procyanidins as the strong determinants of the anti-tumor activity. This suggestion is also supported by the fact that neutral PLE extractions have better cytotoxicity than acidic extracts, as discussed before, and according to the ESI-QTOF MS profile, the neutral extracts are particularly abundant in procyanidins compared to acid extractions (Table 2 and Table 3).

Procyanidins association with cytotoxicity against tumoral cell lines have been described previously for human breast adenocarcinomas [40], oral squamous cells [41], prostate carcinomas [42], hepatocellular carcinomas [43,44], and mostly for colorectal carcinomas [45,46,47,48,49,50]. The publications that assessed the cytotoxicity of tumoral cells linked to procyanidins mainly used extracts from sources such as grape seeds and cocoa [41,46,51]. In the case of grape seed procyanidins, extracts incubated with colorectal carcinomas showed a maximum level of cytotoxicity of 33% in HT-29, and 56% in SW-480 cells treated for 48 h with 100 μg/mL [48]. In our study, for a Satsuma skin neutral extract, a 50% cytotoxic activity was observed in SW-620 cells treated with 46.7 μg/mL, and for Methley flesh neutral extract with 60.9 μg/mL, thus exhibiting better results than those grape skin extracts. Another report on a procyanidin extract from a different species, *Bactris guineensis*, showed better cytotoxicity (IC_50_ 16.6 μg/mL) towards SW-620 cells [50] while exhibiting a lower cytotoxicity towards AGS gastric adenocarcinoma cell lines with an IC_50_ of 113.6 μg/mL. Our study shows a better cytotoxicity towards these AGS gastric cancer tumor lines for neutral extracts from Satsuma skins and flesh, as well as from Methley flesh with IC_50_ values of 60.7, 75.0 and 76.3 μg/mL, respectively. In addition to this, studies show the relationship between procyanidins and higher cytotoxicity on human cancer cells [46,51]; these flavan-3-ol sources may display different in vitro bioactivities for various cancer cells, as the data is influenced by their structure and interactions at the biological level.

In respect to selectivity towards the tumoral cell lines (SW-620, and AGS) when compared to the non-tumor cells (Vero), for all acidic and neutral extracts, a larger cytotoxicity effect was observed. In order to quantify this specificity against the cancer cells, Table 4 shows the values of the selectivity index, which is defined as the ratio of the IC50 values of non-tumor cells to cancer cells. Particularly in the neutral extracts, the highest selectivity index corresponds to SW-620 cells, with SI values from 3.8 to 8.2. In the case of AGS cells, the SI values were between 2.7 and 6.6. This selectivity reported for Costa Rican plum cultivars is better than the SI of 4.1 reported for a plum extract which was used to treat breast cancer cells (MDA-MB-435) compared to breast epithelial cells [52]. Higher values of the selectivity index are desirable, and suggest a possible therapeutic potential. Extracts with an SI greater than 3 are considered to have a high selectivity towards cancer cells [53,54]. This selectivity could be associated with an apoptotic activity [54]. The apoptotic effect has been previously described for a plum extract used to treat human hepatoma HepG2 cells [55], and in the literature this effect has also been associated with the presence of procyanidins [50,56].

These findings merit further studies on other cancer cell types, as plums are edible sources of these bioactive polyphenols, and the results suggest these plum extracts—and particularly Satsuma skins and Methley flesh—as strong candidates for future preventive and therapeutic approaches.

### 2.5. Principal Component Analysis for Polyphenolic Extracts of P. domestica

**Figure 9:**
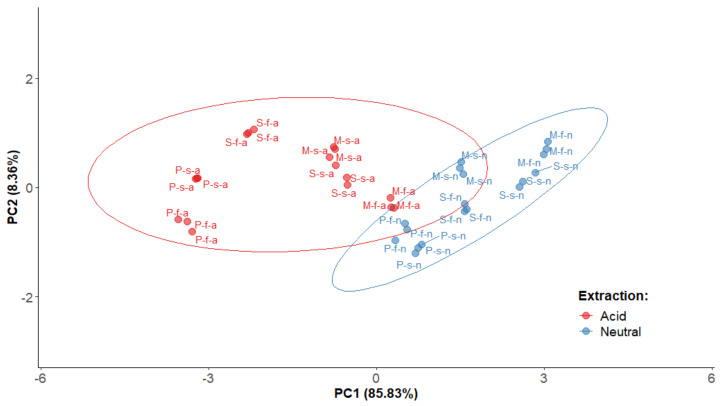
Plane defined by two first principal components (PC1 and PC2) resulting from the principal component analysis (PCA) of PLE polyphenolic extracts of *P. domestica* (*n* = 12). Sample names: C-F-E—Cultivar-Fruit Part-Extraction. Cultivar: M (Methley), P (Pisardii), S (Satsuma). Fruit part: s (skin), f (flesh). Extraction: a (acidic PLE conditions), n (neutral PLE conditions).

In order to summarize the findings, a statistical Principal Component Analysis (PCA) was performed (*n* = 12) considering TPC, DPPH, and ORAC antioxidant values, as well as the cytotoxicity results on gastric adenocarcinoma AGS and colon adenocarcinoma SW620 of polyphenolic extracts. Two components (PC1 and PC2) were obtained (loadings > 0.41), as shown in Figure 9. The first component (PC1) represented 85.83% of the total variance and showed a positive correlation with ORAC, as well as a negative correlation with the DPPH, AGS and SW620 IC_50_ values. The second component (PC2) accounted for 8.36% of the total variance, and was positively correlated to the TP values.

As illustrated in the plane represented by the two components (Figure 9), the skin and flesh extracts are distributed along PC1, and show a less important distribution along PC2, indicating a high variability for their ORAC and DPPH values and cytotoxicity IC_50_ results on AGS and SW620 cancer cell lines. However, a clear trend can be observed for all of the samples obtained under neutral extraction conditions with better values than the ones obtained under acidic extraction conditions. Some samples have particularly low values in PC1—for instance, Pisardii cultivar skins and flesh extracts under PLE acid conditions—exhibiting poorer antioxidant and anticancer results. Meanwhile, Methley flesh and Satsuma skin neutral extracts show the highest PC1, which agrees with the results discussed in the previous sections, accounting for their especially high antioxidant and cytotoxic activity among all twelve extracts. Furthermore, our findings showed that all of the acid extracts are grouped with low scores in PC1 and, in contrast, neutral samples are more homogenously grouped in the higher score in PC1, with a greater antioxidant capacity and cytotoxic activity towards AGS and SW620 tumor cells, independently of the part or cultivar involved. In sum, as discussed previously, the results show that *P. domestica* extracts obtained under Neutral PLE conditions, particularly from the flesh of the Methley cultivar and from the skins of the Satsuma cultivar, are promising substrates for further studies to assess their actual benefits on human health.

## 3. Materials and Methods

### 3.1. Materials, Reagents and Solvents

*P. domestica* fruits of the Methley, Pisardii and Satsuma cultivars were acquired in a ripe state from Frutalcoop local producers in Los Santos, Costa Rica. The cultivars were confirmed with the support of the Costa Rican National Herbarium, and vouchers were deposited there. The reagents, such as fluorescein, 2,2-azobis(2-amidinopropane) dihydrochloride (AAPH), 2,2-diphenyl-1-picrylhidrazyl (DPPH), Trolox, gallic acid, Amberlite XAD-7 resin, fetal bovine serum, glutamine, penicillin, streptomycin, amphotericin B, and trypsin–EDTA, were provided by Sigma-Aldrich (St. Louis, MO, USA). The human gastric adenocarcinoma cell line AGS, human colorectal adenocarcinoma SW 620 and monkey normal epithelial kidney cells Vero were obtained from the American Type Culture Collection (ATCC, Rockville, MD), while the solvents—such as acetone, chloroform and methanol—were purchased from Baker (Center Valley, PA, USA), and the DMSO was acquired from Sigma-Aldrich (St. Louis, MO, USA).

### 3.2. Phenolic Extracts from Prunus domestica Fruits

The *P. domestica* fruits were rinsed in water, peeled, and both the skin and flesh material were freeze-dried in a Free Zone −105 °C, 4.5 L, Cascade Benchtop Freeze Dry System (Labconco, Kansas, MO, USA). The freeze-dried material was preserved at −20 °C until extraction. The freeze-dried samples were extracted under Pressurized Liquid Extraction (PLE) conditions in a Dionex™ ASE™ 150 Accelerated Solvent Extractor (Thermo Scientific™, Walthman, MA, USA) using methanol:water (70:30) as the solvent in a 34 mL cell, at 40 °C, under neutral conditions or using 0.1% formic acid. Next, the extract was evaporated under vacuum to eliminate the methanol, and the aqueous phase was washed with ethyl acetate and chloroform to remove the less-polar compounds. Afterwards, the aqueous extract was evaporated under vacuum to eliminate organic solvent residues, and was eluted (2 mL/min) in an Amberlite XAD7 column (150 mm × 20 mm), starting with 300 mL of water to remove the sugars, and then with 200 mL each of methanol and water (80:20) and pure methanol to obtain the polyphenols. Finally, the enriched extract was obtained after evaporating to dryness using a Buchi™ 215 (Flawil, Switzerland) rotavapor.

### 3.3. Total Phenolic Content

The polyphenolic content was determined as previously reported [57] by a modification of the Folin–Ciocalteu (FC) method [58], for which the reagent is composed of a mixture of phosphotungstic and phosphomolybdic acids. Each sample was dissolved in MeOH (0.1% HCl) and combined with 0.5 mL FC reagent. Afterwards, 10 mL Na_2_CO_3_ (7.5%) was added and the volume was completed to 25 mL with water. The blanks were prepared in a similar way, but using 0.5 mL MeOH (0.1% HCl) instead of the sample. The mixture was left standing in the dark for 1 h, and then the absorbance was measured at 750 nm. The values obtained were extrapolated in a gallic acid calibration curve. The total phenolic content was expressed as mg gallic acid equivalents (GAE)/g sample. The analyses were performed in triplicate.

### 3.4. UPLC-ESI-MS Analysis

The UPLC-MS system used to analyze the composition of the *P. domestica* extracts consisted of an Xevo G2-XS QTOF (Waters, UK) coupled with an AQUITY H Class UPLC system with a quaternary pump. The ESI source parameters were set to a capillary voltage of 2 kV, a sampling cone of 20 eV, a source temperature of 150 °C, and a source offset of 10 °C. The desolvation temperature was set to 450 °C, the cone gas flow was set to 0 L/h, and the desolvation gas flow was set to 900 L/h.

The measurement was performed in MSe negative mode using an acquisition mass range from 100 *m*/*z* to 2000 *m*/*z* and a scan rate of 0.5 s, where the fragmentation was carried out using Independent Data Acquisition for all of the eluting compounds, with a collision energy ramp from 20 V to 30 V for the high-energy function. Instrument calibration was applied in the mass range of the measurement with sodium formate. Lock mass correction was applied directly to the measurement using a leucine enkephalin infusion measured every 30 s during the run.

The separation was carried out on a Luna RP-C18 column (150 mm × 4.6 mm i.d. × 4 µm, Phenomenex, Torrance, CA) with a pre-column filter (Phenomenex, Torrance, CA, USA). In total, 1 uL of the sample was injected at a flow rate of 0.3 mL/min using a gradient of Mobile phase A and B consisting of a combination of 0.1% formic acid in water, *v*/*v,* and 0.1% formic acid in acetonitrile, *v*/*v*, respectively. The gradient was from 4% to 20% B (*v*/*v*) at 20 min, to 35% B at 35 min, and to 100% B at 40 min, and was held at 100% B to 45 min.

### 3.5. DPPH Radical-Scavenging Activity

The DPPH evaluation was performed as previously reported [59], and was expressed as IC_50_ (µg/mL), which is the amount of sample required to reach the 50% radical-scavenging activity. Briefly, a solution of 2,2-diphenyl-1-picrylhidrazyl (DPPH) (0.25 mM) was prepared using methanol as the solvent. Next, 0.5 mL of this solution was mixed with 1 mL of the extract at different concentrations, and incubated at 25 °C in the dark for 30 min. The DPPH absorbance was measured at 517 nm. Blanks were prepared for each concentration. The percentage of the radical-scavenging activity of the sample was plotted against its concentration to calculate IC_50_ (µg/mL). The samples were analyzed in three independent assays. Trolox was used as the reference antioxidant agent. A methanolic solution at different concentrations was used instead of the samples along the described procedure. Trolox IC_50_ (µg/mL) was converted to mmol/mL using the Trolox molecular weight (250.29 mg/mmol), and was then divided by the IC_50_ of each sample in order to express the DPPH results as mmol of Trolox equivalents (TE)/g extract.

### 3.6. ORAC Antioxidant Activity

The ORAC (Oxygen Radical Absorbance Capacity) antioxidant activity was determined following a method previously described [60,61] using fluorescein as a fluorescence probe. The reaction was performed in 75 mM phosphate buffer (pH 7.4) at 37 °C. The final assay mixture consisted of AAPH (12 mM), fluorescein (70 nM), and either Trolox (1–8 µM) or the extract at different concentrations. The fluorescence was recorded every minute for 98 min in black 96-well untreated microplates (Nunc, Denmark), using a Polarstar Galaxy plate reader (BMG Labtechnologies GmbH, Offenburg, Germany) with 485-P excitation and 520-P emission filters. Fluostar Galaxy software version 4.11-0 (BMG Labtechnologies GmbH, Offenburg, Germany) was used to measure the fluorescence. Fluorescein was diluted from a stock solution (1.17 mM) in 75 mM phosphate buffer (pH 7.4), while the AAPH and Trolox solutions were freshly prepared. All of the reaction mixtures were prepared in duplicate, and three independent runs were completed for each extract. The fluorescence measurements were normalized to the curve of the blank (no antioxidant). From the normalized curves, the area under the fluorescence decay curve (AUC) was calculated as:

(1) $$\mathrm{AUC}=1+\overset{i=98}{\underset{i=1}{\sum }}\int_{i}/\int_{0}$$

where *f*_0_ is the initial fluorescence reading at 0 min, and *f*i is the fluorescence reading at time *i*. The net AUC corresponding to a sample was calculated as follows:

(2) $$\mathrm{Net}\;\mathrm{AUC}={\mathrm{AUC}}_{\mathrm{antioxidant}}-{\mathrm{AUC}}_{\mathrm{blank}}$$

The regression equation between the net AUC and antioxidant concentration was calculated. The ORAC value was estimated by dividing the slope of the latter equation by the slope of the Trolox line obtained for the same assay. The final ORAC values were expressed as the mmol of Trolox equivalents (TE)/g of the phenolic extract.

### 3.7. Evaluation of the Cytotoxicity of the Extracts

#### 3.7.1. Cell Culture

The human gastric adenocarcinoma cell line AGS, the human colorectal adenocarcinoma SW 620, and monkey normal epithelial kidney cells Vero were grown in minimum essential Eagle’s medium (MEM) containing 10% fetal bovine serum (FBS) in the presence of 2 mmol/L glutamine, 100 IUmL^−1^ penicillin, 100 μg/mL streptomycin and 0.25 μg/mL amphotericin B. The cells were grown in a humidified atmosphere containing 5% CO_2_ at 37 °C, and were sub-cultured by detaching them with trypsin–EDTA solution at about 70–80% confluence. For the experiments, 100 μL of a cell suspension of 1.5 × 10^5^ cells/mL was seeded overnight into 96-well plates. The cells were further exposed for 48 h to various concentrations of extracts (50 μL), dissolved in DMSO, and diluted with cell culture medium to final concentrations between 15–500 μg/mL. The DMSO concentrations used in the experiments were below 0.1% (*v*/*v*), and the control cultures were prepared with the addition of DMSO (the vehicle control).

#### 3.7.2. Assessment of the Cytotoxicity by the MTT Assay

After incubation for 48 h, MTT assays were performed to evaluate the cytotoxicity. Briefly, the medium was eliminated, the cells were washed twice with 100 µL PBS and incubated with 100 µL MTT solution (3-(4,5-dimethylthiazolyl-2)-2,5-diphenyltetrazolium bromide, 5 mg/mL in a cell culture medium) for 2 h at 37 °C. The formazan crystals formed were dissolved in 100 µL 95% ethanol, and the absorbance was read at 570 nm in a microplate reader. Dose–response curves were established for each extract, and the concentration which was enough to reduce the cell viability by 50% (IC _50_) was calculated.

In order to evaluate whether the cytotoxicity activity was specific against the cancer cells, a selectivity index (SI) was determined. This index is defined as the ratio of the IC_50_ values of normal epithelial kidney cells (Vero) to cancer cells (AGS or SW620).

### 3.8. Statistical Analysis

One-way analysis of variance (ANOVA) followed by Tukey’s post hoc test was applied to the TPC, DPPH, ORAC and cytotoxicity results, and the differences were considered significant at *p* < 0.05. Two-way analyses of variance (ANOVA) were applied to evaluate both factors, i.e., the part of the fruit and the extraction conditions used. In order to evaluate whether the total phenolic contents (TPC) contribute to the antioxidant activity evaluated with the DPPH and ORAC methodologies, a correlation analysis was carried out as well as the cytotoxicity assays. Finally, a Principal Component Analysis (PCA) was performed using R (version x64 4.1.1) as the statistical program.

## 4. Conclusions

This paper reports valuable information on the profile of the phenolic-enriched extracts of the main commercial cultivars of *P. domestica* in Costa Rica, using UPLC-ESI-QTOF MS techniques, showing a total of 306 compounds characterized in the twelve extracts obtained under acidic and neutral PLE conditions for the skins and flesh of the Methley, Pisardii and Satsuma cultivars. Extracts obtained under Neutral PLE conditions exhibited a higher number of polyphenols for the skins and flesh in all three cultivars, and indicated a higher and more diverse number than the cultivars from other countries reported in the literature. The PCA results align with the higher values obtained under neutral PLE conditions for TPC, DPPH and ORAC antioxidant activities, and with the cytotoxicity towards AGS and SW 620 adenocarcinoma cell lines. Furthermore, these cytotoxicity values show a high significant negative correlation with procyanidins (r= −0.868 and r= −0.855 respectively). As discussed, the relationships of these metabolites and their antioxidant capacity with health benefits [62], including anti-inflammatory effects and cancer chemo-preventive properties, suggest their potential application as functional ingredients [63]. In sum, the overall results—and particularly those obtained for Methley flesh and Satsuma skins—suggest the potential of these Neutral PLE extracts to carry further research, for instance, using other cancer cell lines and antioxidant models [64], towards a comprehensive approach to their potential application in the nutraceutical industry.

## Figures and Tables

**Table 1:** Total phenolic content for plum samples under acid and neutral extraction conditions.

Sample	Total Phenolic Content (TPC) (mg/g) ^1,2,3^	
	Acid Extraction	Neutral Extraction
Methley		
Skin	539.3 ^a,^* ± 8.55	669.1 ^a,#^ ± 11.2
Flesh	567.7 ^b,^* ± 8.40	801.6 ^b,#^ ± 14.2
Pisardii		
Skin	438.5 ^c,^* ± 5.08	560.5 ^cd,#^ ± 8.34
Flesh	319.9 ^d,^* ± 3.86	538.5 ^d,#^ ± 13.9
Satsuma		
Skin	513.9 ^e,^* ± 4.10	685.0 ^a,#^ ± 14.1
Flesh	477.2 ^f,^* ± 10.3	596.1 ^c,#^ ± 4.89

^1^ mg of gallic acid equivalent (GAE)/g extract. ^2^ Values are expressed as the mean ± standard deviation (S.D.). ^3^ Different superscript letters in the same column or different superscript signs in the same row indicate that the differences are significant at *p* < 0.05 using one-way analysis of variance (ANOVA) with a Tukey post hoc.

**Table 2:** Profile of phenolic compounds identified by UPLC-ESI-QTOF MS analysis for plum skin and flesh samples under acidic and neutral PLE conditions.

#	Tentative Identification	[M-H]^−^	tR (min)	MS2 Fragments	Formula
Hydroxycinnamic acids and derivatives					
1	Caffeoylferuloylquinic acid (I of II)	529.1365	2.89	[529]: 353, 367, 191, 179	C26H25O12
3	*p*-Coumaroyl hexoside (I of III)	325.0917	4.42	[325]: 145, 163, 187	C15H17O8
5	Caffeoyl hexoside	341.0884	7.13	[341]: 161, 179	C15H17O9
6	Caffeoylquinic acid isomer	353.0869	7.56	[353]: 191, 145	C16H17O9
7	*p*-Coumaroyl hexoside (II of III)	325.0917	7.96	[325]: 145, 163, 187	C15H17O8
12	*p*-Coumaroyl hexoside (III of III)	325.0917	9.98	[325]: 145	C15H17O8
13	Feruloylquinic acid isomer (I of III)	367.1021	10.23	[367]: 161, 134	C17H19O9
15	Feruloylquinic acid isomer (II of III)	367.1021	11.45	[367]: 193, 134	C17H19O9
19	Caffeoylferuloylquinic acid (II of II)	529.1365	12.8	[529]: 353, 367, 191, 179	C26H25O12
21	*p*-coumaroylquinic acid isomer (I of II)	337.0919	14.78	[337]:173	C16H17O8
22	*p*-coumaroylquinic acid isomer (II of II)	337.0919	15.29	[337]:173	C16H17O8
23	Feruloylquinic acid isomer (III of III)	367.1021	15.65	[367]: 193, 134	C17H19O9
24	Methyl-*p*-Coumaroylquinic acid (I of II)	351.1082	16.99	[351]: 177, 293, 235, 191, 133	C17H19O8
25	Di-O-acetyl-O-*p*-coumaroylsucrose (I of II)	571.1675	17.44	[571]: 553, 529, 511, 487, 307	C25H31O15
29	Methyl-*p*-Coumaroylquinic acid (II of II)	351.1082	20.55	[351]:177, 293, 235, 191, 133	C17H19O8
30	Di-O-acetyl-O-*p*-coumaroylsucrose (II of II)	571.1675	21.03	[571]: 529, 511, 307, 175	C25H31O15
Flavonoids					
4	Kaempferol-deoxyhexoside	447.0913	5.89	[447]: 300, 285	C21H19O11
18	Quercetin-deoxyhexoside (I of II)	447.0913	12.57	[447]: 300, 301	C21H19O11
31	Quercetin-hexoside (I of III)	463.0901	21.95	[463]: 300, 301	C21H19O12
32	Quercetin-rutinoside (I of II)	609.1488	22.86	[609]:300, 301	C27H29O16
33	Quercetin-hexoside (II of III)	463.0875	23.89	[463]: 300, 301	C21H19O12
34	Quercetin-hexoside (III of III)	463.0875	25.46	[463]: 300, 301	C21H19O12
35	Quercetin-pentoside (I of II)	433.0764	25.95	[433]: 300, 301	C20H17O11
36	Quercetin-pentosylpentoside	565.1208	26.24	[565]: 300, 301	C25H25O15
37	Quercetin-pentoside (II of II)	433.0769	27.5	[433]: 300, 301	C20H17O11
38	Quercetin-rutinoside isomer (II of II)	609.1488	27.86	[609]:300, 301	C27H29O16
39	Quercetin-deoxyhexoside (II of II)	447.0913	28.68	[447]: 300, 301	C21H19O11
40	Quercetin-acetylhexoside	505.0974	29.09	[505]: 300, 301	C23H21O13
41	Quercetin 3-O-p-coumaroyl acetylhexoside	651.1553	34.27	[651]: 633, 505, 487, 301, 300	C32H27O15
42	Quercetin	301.0353	37.25	[301]: 283, 273, 257, 255, 179, 151	C15H9O7
Flavan-3-ols					
8	Procyanidin dimer A (I of II)	575.1183	8.05	[575]: 285, 289, 423, 449	C30H23O12
9	Procyanidin B-type trimer (I of III)	865.2014	8.28	[865]: 287, 289, 575, 577, 695, 713, 739	C45H37O18
10	Catechin	289.0704	8.81	[289]: 245, 271	C15H13O6
11	Procyanidin B-type dimer (I of II)	577.1332	9.27	[577]: 287, 289, 407, 425, 451, 559	C30H25O12
14	Procyanidin B-type trimer (II of III)	865.2014	11.25	[865]: 287, 289, 575, 577, 695, 713, 739	C45H37O18
16	Procyanidin B-type tetramer	1153.2579	11.86	[1153]: 287, 289, 575, 577, 863, 865, 983, 1001, 1027, 1135	C60H49O24
17	Epicatechin	289.0704	12.16	[289]: 245, 271	C15H13O6
20	Procyanidin A-type trimer	863.1790	13.71	[863]: 287, 575, 711	C45H35O18
26	Procyanidin B-type trimer (III of III)	865.1993	18.28	[865]: 287, 289, 575, 577, 695, 713, 739	C45H37O18
27	Procyanidin A-type dimer	575.1183	18.74	[575]: 285, 289, 423, 449	C30H23O12
28	Procyanidin B-type dimer (II of II)	577.1332	19.89	[577]: 287, 289, 407, 425, 451, 559	C30H25O12
Other acids					
2	Quinic acid	191.0554	3.08	[191]: 173, 127	C7H11O6

**Table 3:** DPPH and ORAC antioxidant activities for plum samples under acid and neutral extraction conditions.

Sample	DPPH IC_50_ (μg/mL) ^1,2^		ORAC (mmol TE/g Extract)^1,3^	
	Acid Extraction	Neutral Extraction	Acid Extraction	Neutral Extraction
Methley				
Skin	7.59 ^ab,^* ± 0.28	5.79 ^a,#^ ± 0.13	9.23 ^a,^^ ± 0.31	10.91 ^b,&^ ± 0.07
Flesh	6.81 ^bc,^* ± 0.24	4.39 ^b,#^ ± 0.15	8.74 ^ab,^^ ± 0.13	12.22 ^a,&^ ± 0.17
Pisardii				
Skin	12.72 ^d,^* ± 0.20	8.57 ^c,#^ ± 0.24	7.45 ^cd,^^ ± 0.11	9.71 ^c,&^ ± 0.31
Flesh	12.44 ^d,^* ± 0.35	8.17 ^c,#^ ± 0.25	7.04 ^d,^^ ± 0.13	9.76 ^c,&^ ± 0.44
Satsuma				
Skin	6.73 ^c,^* ± 0.17	4.85 ^b,#^ ± 0.04	8.53 ^b,^^ ± 0.18	12.55 ^a,&^ ± 0.40
Flesh	8.40 ^a,^* ± 0.20	4.96 ^b,#^ ± 0.15	7.79 ^c,^^ ± 0.15	10.05 ^bc,&^ ± 0.07

^1^ Values are expressed as the mean ± S.D. ^2,3^ Different superscript letters in the same column or different superscript signs in the same row indicate that the DPPH IC50 or ORAC differences are significant at *p* < 0.05 using one-way analysis of variance (ANOVA) with a Tukey post hoc statistical test.

**Table 4:** Cytotoxicity of *P. domestica* samples extracted under acidic and neutral conditions for gastric (AGS) and colon (SW-620) carcinoma cells, as well non-tumoral cells (Vero).

**Sample**	**AGS**	**SW620**	**Vero**
	**IC_50_ (μg/mL) ^1,2^ (SI) ^3^**		
	**Acid Extraction**		
Methley			
Skin	403 ^a,^* ± 13 (1.2)	318 ^a,#^ ± 5 (1.6)	>500 ^a,&^
Flesh	196 ^b,*^ ± 9 (2.6)	161 ^b,#^ ± 5 (3.1)	>500 ^a,&^
Pisardii			
Skin	>500^c^	>500 ^c^	>500 ^a^
Flesh	>500 ^c,^*	387 ^d,#^ ± 24 (1.3)	>500 ^a,^*
Satsuma			
Skin	340 ^d,^* ± 29 (1.5)	255 ^e,#^ ± 16 (2.0)	>500 ^a,&^
Flesh	>500 ^c^	>500 ^c^	>500 ^a^
	**AGS**	**SW620**	**Vero**
	**IC_50_ (μg/mL) ^1,2^ (SI) ^3^**		
	**Neutral Extraction**		
Methley			
Skin	186 ^a,^* ± 7 (2.7)	122 ^a,#^ ± 7 (4.1)	>500 ^a,&^
Flesh	76.3 ^b,^* ± 6.8 (6.6)	60.9 ^bc,^* ± 4.1 (8.2)	>500 ^a,#^
Pisardii			
Skin	83.1 ^b,^* ± 5.2 (6.0)	67.9 ^b,#^ ± 2.4 (7.4)	>500 ^a,&^
Flesh	125 ^c,^* ± 10 (4.0)	132 ^a,^* ± 3 (3.8)	>500 ^a,#^
Satsuma			
Skin	60.7 ^b,^* ± 4.6 (6.2)	46.7 ^c,^* ± 1.5 (8.1)	378 ^b,#^ ± 33
Flesh	75.0 ^b,^* ± 5.1 (6.6)	73.0 ^b,^* ± 4.1 (6.8)	>500 ^a,#^

^1^ Values are expressed as the mean ± D.E. ^2^ Different superscript letters in the same column or different superscript signs in the same row indicate that the IC_50_ differences are significant at *p* < 0.05 using a one-way analysis of variance (ANOVA) with a Tukey post hoc statistical test. ^3^ (SI) = Selectivity Index.

## Data Availability

The data presented in this study are available within this article and Supplementary Material.

## Supplementary Materials

Figure S1: Caffeoyl-feruloyl-quinic acid fragmentation pathway, Figure S2: Structure and fragmentation of di-O-acetyl-O-p-coumaroylsucrose. Figure S3: Quercetin fragmentation pathway. Figure S4: Fragmentation pathway of A-type procyanidin dimer showing the products formed by heterocyclic ring fussion (HRF), quinone methide (QM) and retro-Diels–Alder (RDA) reactions, Figure S5: Fragmentation pathway of B-type procyanidin dimer showing the products formed by heterocyclic ring fussion (HRF), quinone methide (QM) and retro-Diels–Alder (RDA) reactions. Table S1: Profile of phenolic compounds identified by UPLC-ESI-QTOF MS analysis, for plum skin (S) and flesh (F) samples under PLE neutral and acidic conditions.

## Sample Availability

Samples of the compounds are not available from the authors.
