# Supplementary material for: Polyphenolic QTOF-ESI MS Characterization and the Antioxidant and Cytotoxic Activities of Prunus domestica Commercial Cultivars from Costa Rica
Source: Molecules. 2021 Oct 27;26(21):6493. doi: 10.3390/molecules26216493 (PMC8588404; doi:10.3390/molecules26216493)
Supplement: Supplementary file 1 [file molecules-26-06493-s001.zip › molecules-1425996-supplementary.pdf]

Table S1. Profile of phenolic compounds identified by UPLC-ESI-QTOF MS analysis for plum skin (S) and flesh (F) samples under PLE neutral and acidic conditions.

#	[M-H]-	tR (min)	MS2 fragments	Formula	Satsuma (neutral)		Methley (neutral)		Pisardii (neutral)		Satsuma (acid)		Methley (acid)		Pisardii (acid)	
					S	F	S	S	F	S	S	F	S	S	F	S
Hydroxycinnamic acids and derivatives																
1	529.1365	2.89	[529]: 353, 367, 191, 179	C26H25O12		x	x			x						
3	325.0917	4.42	[325]: 145, 163, 187	C15H17O8							x		x			
5	341.0884	7.13	[341]: 161, 179	C15H17O9	x		x		x	x	x	x	x	x	x	x
6	353.0869	7.56	[353]: 191, 145	C16H17O9	x		x	x	x	x	x	x	x	x	x	x
7	325.0917	7.96	[325]: 145, 163, 187	C15H17O8	x		x	x		x						
12	325.0917	9.98	[325]: 145	C15H17O8	x	x	x		x	x	x	x	x	x	x	x
13	367.1021	10.23	[367]: 161 , 134	C17H19O9	x	x	x		x	x			x		x	
15	367.1021	11.45	[367]: 193 , 134	C17H19O9	x	x	x	x	x	x	x	x	x	x	x	x
19	529.1365	12.8	[529]: 353, 367, 191, 179	C26H25O12			x			x	x	x	x	x	x	x
21	337.0919	14.78	[337]:173	C16H17O8	x				x	x	x	x	x			x
22	337.0919	15.29	[337]:173	C16H17O8	x					x	x	x				
23	367.1021	15.65	[367]: 193 , 134	C17H19O9	x	x	x	x	x	x						x
24	351.1082	16.99	[351]: 177 , 293 , 235 , 191, 133	C17H19O8	x				x							x
25	571.1675	17.44	[571]: 553, 529, 511, 487, 307	C25H31O15			x		x		x		x	x	x	x
29	351.1082	20.55	[351]:177 , 293 , 235 , 191, 133	C17H19O8	x		x	x		x	x					
30	571.1675	21.03	[571]: 529, 511, 307, 175	C25H31O15			x	x			x	x	x	x	x	x
Flavonoids																
4	447.0913	5.89	[447]: 300, 285	C21H19O11	x	x	x	x	x	x	x	x	x	x	x	x
18	447.0913	12.57	[447]: 300, 301	C21H19O11	x	x		x	x		x	x	x	x	x	
31	463.0901	21.95	[463]: 300, 301	C21H19O12	x		x				x		x	x	x	
32	609.1488	22.86	[609]:300, 301	C27H29O16	x	x	x		x	x	x	x	x	x	x	x
33	463.0875	23.89	[463]: 300, 301	C21H19O12	x	x	x	x	x	x	x	x	x	x	x	x
34	463.0875	25.46	[463]: 300, 301	C21H19O12	x		x				x		x			

35	433.0764	25.95	[433]: 300, 301	C20H17O11			x	x			x	x	x	x	x	x
36	565.1208	26.24	[565]: 300, 301	C25H25O15	x						x	x	x	x	x	
37	433.0769	27.5	[433]: 300, 301	C20H17O11	x		x			x	x	x	x	x		x
38	609.1488	27.86	[609]:300, 301	C27H29O16			x	x						x	x	
39	447.0913	28.68	[447]: 300, 301	C21H19O11	x		x		x		x		x	x	x	
40	505.0974	29.09	[505]: 300, 301	C23H21O13	x						x		x		x	
41	651.1553	34.27	[651]: 633, 505, 487, 301, 300	C32H27O15									x	x	x	
42	301.0353	37.25	[301]: 283, 273, 257, 255, 179, 151	C15H9O7	x	x	x	x	x		x		x			
Flavan-3-ols																
8	575.1183	8.05	[575]: 285, 289, 423, 449	C30H23O12								x		x		
9	865.2014	8.28	[865]: 287, 289, 575, 577, 695, 713, 739	C45H37O18					x		x	x	x	x	x	x
10	289.0704	8.81	[289]: 245, 271	C15H13O6	x	x	x	x	x	x	x	x	x	x	x	x
11	577.1332	9.27	[577]: 287, 289, 407, 425, 451, 559	C30H25O12	x		x	x		x	x	x	x	x	x	x
14	865.2014	11.25	[865]: 287, 289, 575, 577, 695, 713, 739	C45H37O18			x				x	x	x	x	x	x
16	1153.2579	11.86	[1153]: 287, 289, 575, 577, 863, 865, 983, 1001, 1027, 1135	C60H49O24							x	x	x	x	x	
17	289.0704	12.16	[289]: 245, 271	C15H13O6	x	x	x	x	x	x	x	x	x	x	x	x
20	863.1790	13.71	[863]: 287, 575, 711	C45H35O18	x		x				x	x	x			
26	865.1993	18.28	[865]: 287, 289, 575, 577, 695, 713, 739	C45H37O18									x			x
27	575.1183	18.74	[575]: 285, 289, 423, 449	C30H23O12	x	x	x	x	x	x	x	x	x	x	x	x
28	577.1332	19.89	[577]: 287, 289, 407, 425, 451, 559	C30H25O12	x		x	x			x	x	x	x	x	
Other acids																
2	191.0554	3.08	[191]: 173, 127	C7H11O6	x	x	x	x	x	x	x	x	x	x	x	x

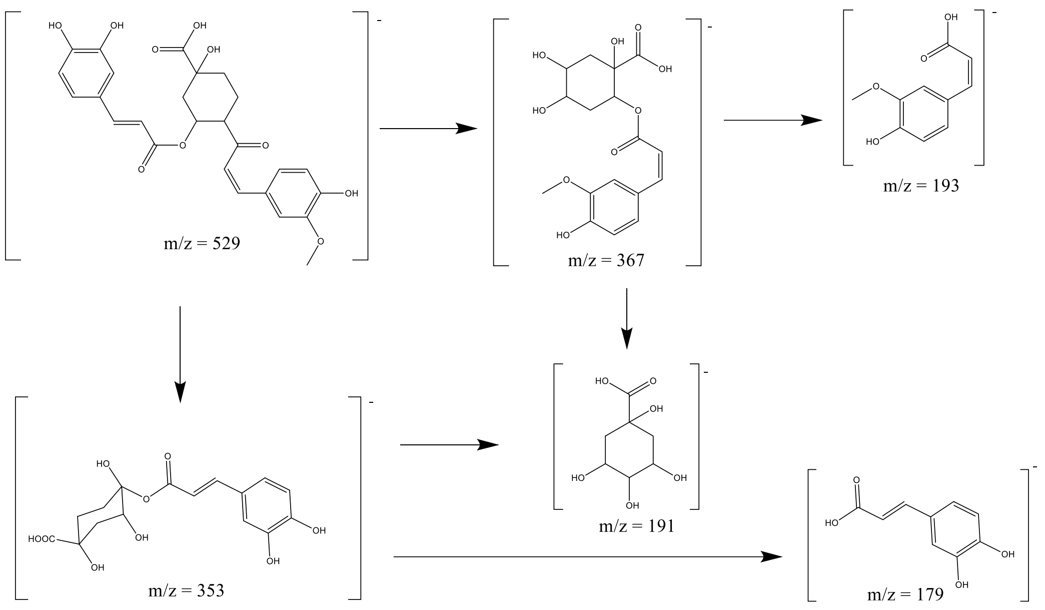

Figure S1. Caffeoyl-feruloyl-quinic acid fragmentation pathway.

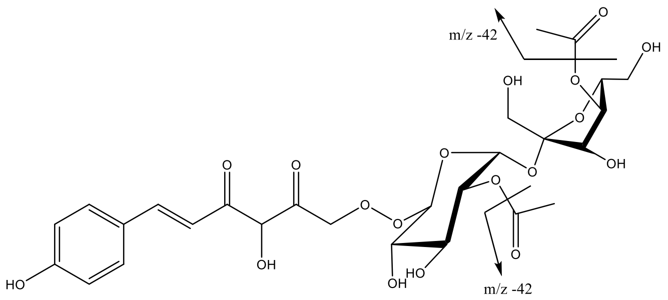

Figure S2. Structure and fragmentation of di-O-acetyl-O-*p*-coumaroylsucrose.

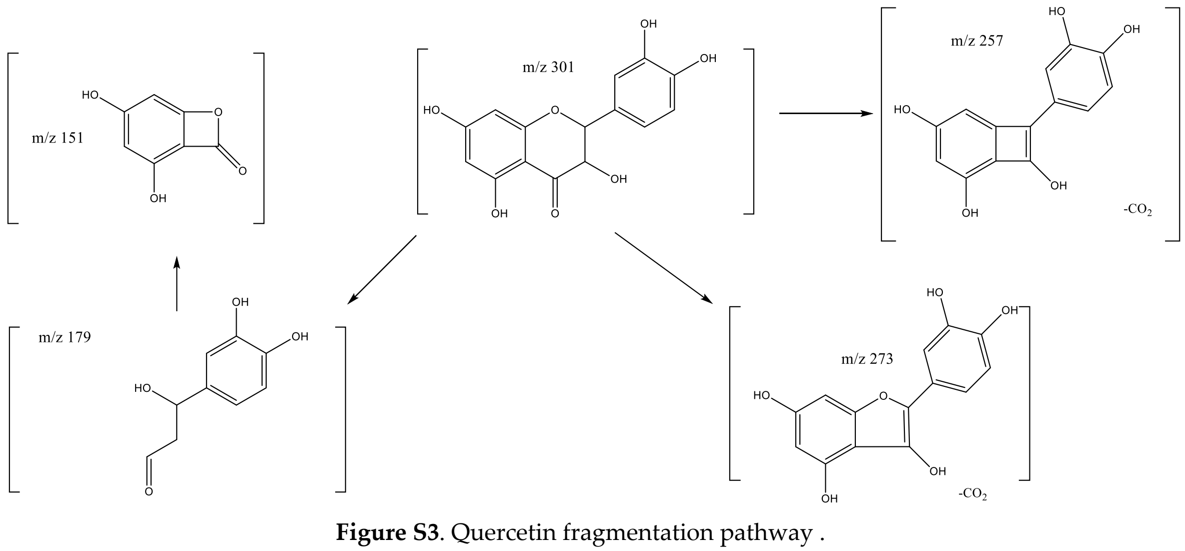

Figure S3. Quercetin fragmentation pathway .

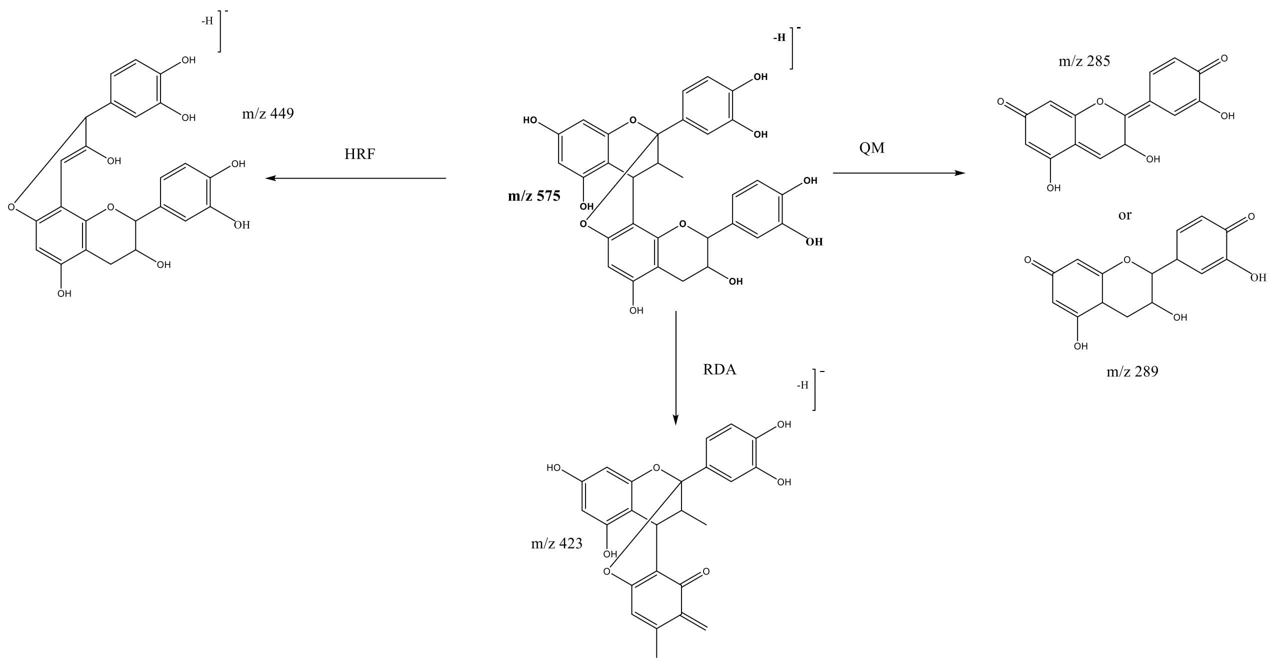

Figure S4. Fragmentation pathway of A-type procyanidin dimer showing the products formed by heterocyclic ring fussion (HRF), quinone methide (QM) and retro-Diels–Alder (RDA) reactions.

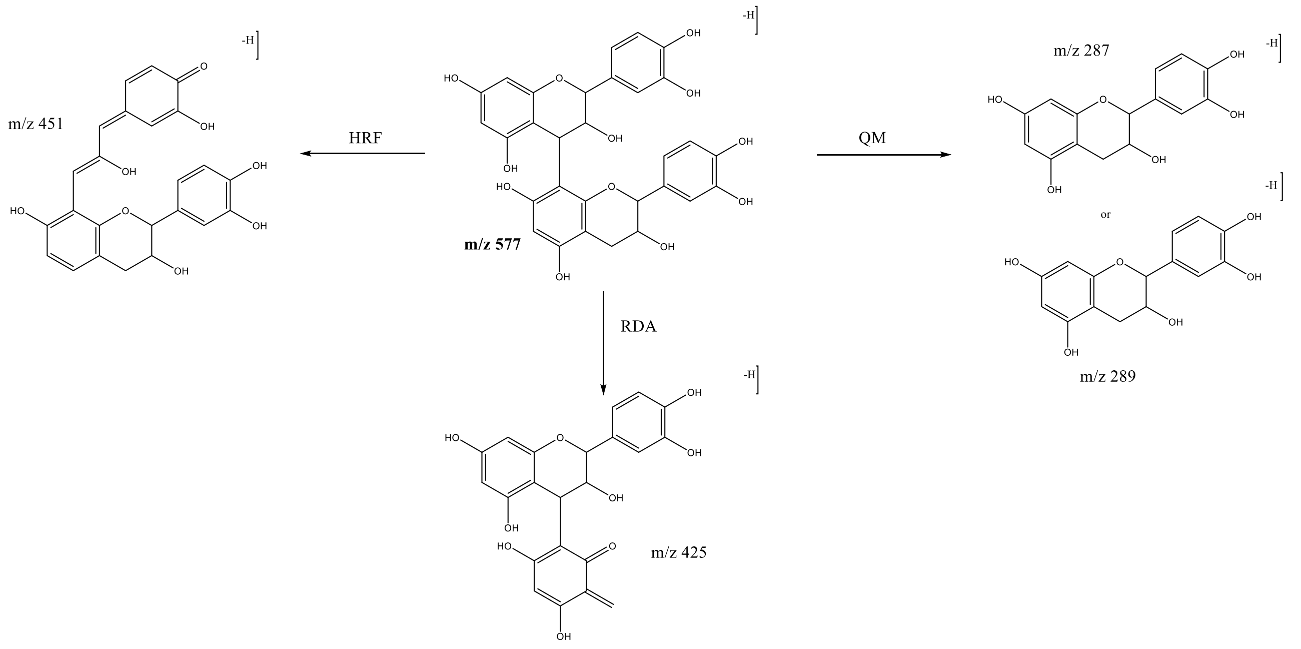

Figure S5. Fragmentation pathway of B-type procyanidin dimer showing the products formed by heterocyclic ring fussion (HRF), quinone methide (QM) and retro-Diels–Alder (RDA) reactions.
